# Avulsion of both posterior meniscal roots associated with acute rupture of the anterior cruciate ligament

**DOI:** 10.1007/s10195-014-0302-2

**Published:** 2014-06-28

**Authors:** Pier Paolo Mariani, Germano Iannella, Guglielmo Cerullo, Marco Giacobbe

**Affiliations:** 1 Università Roma 4-Foro Italico, Piazza L. de Bosis 5, 00136 Rome, Italy; 2 Clinic “Villa Stuart”, via Trionfale 5952, 00136 Rome, Italy

**Keywords:** Meniscus, Meniscal root, ACL, Knee

## Abstract

A rare case of acute avulsion of both posterior meniscal roots concomitant with an acute anterior cruciate ligament (ACL) tear in a professional soccer player is described. While avulsion of the lateral meniscal root has been extensively reported in association with ACL injuries, medial root avulsion has never been reported in association with acute ACL. A review of the video documentation of the match accident revealed the exact mechanism of injury was a forceful external rotation of the standing limb.

## Introduction

Root tears are a subset of meniscal injuries, which have become increasingly recognized as a cause of pain and impaired mobility. The root serves as the anchor point for the menisci. Occurring on either the medial or lateral meniscus, root tears refer to a radial tear or avulsion at the posterior horn attachment to the bone. Both radial tear and posterior horn avulsion defunction the menisci as load-bearing structures, with increasing local contact pressure and premature onset of knee arthritis [9]. Because the medial and lateral menisci differ in anatomy and biomechanics, the pathogenesis of posterior root avulsions is also different. Tearing of the lateral posterior meniscal root is traumatic and always associated with anterior cruciate ligament (ACL) injury [1, 22], while the medial posterior root [2, 8, 10, 19] is prone to chronic degenerative meniscal disease.

To date, only one case [15] has been reported of radial tear of both roots detected two years after an ACL injury. The authors hypothesised a traumatic origin for avulsion of the lateral meniscus and a degenerative origin for the medial meniscus.

Here, we describe a rare case of avulsion of both posterior roots in association with an acute ACL tear in a professional soccer player. A review of the video documentation of the match accident revealed the exact mechanism of injury.

## Case report

A 20-year-old professional soccer player reported sustaining a forceful rotatory left knee injury during an official match of the Italian Second Division. The dynamics of the accident could be clearly followed on the video recording of the match. While the player was standing with one foot fixed on the field and the contralateral limb elevated for shooting the ball, a player from the opponent team collided into the elevated limb, causing a forceful external rotation of the standing limb.

**Fig. 1:**
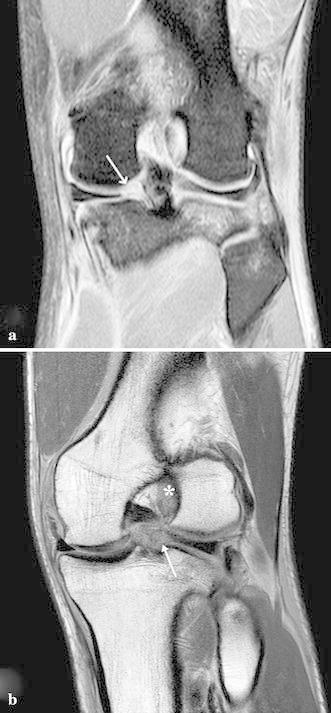
**a** Preoperative coronal TSE-fat saturated MR image shows the ACL tear and an area of heterogeneous intrameniscal signal intensity of both meniscal roots with a void at the posterior attachment site of both menisci (*arrow*). **b** Preoperative coronal oblique 30° MR image. The *arrow* shows absence of tibial insertion of posterior lateral root. The *asterisk* shows hyperintensity signal at femoral ACL insertion

**Fig. 2:**
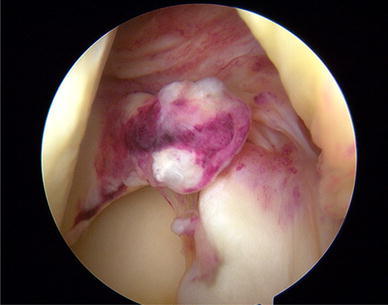
Arthroscopic view of acute medial posterior root avulsion

**Fig. 3:**
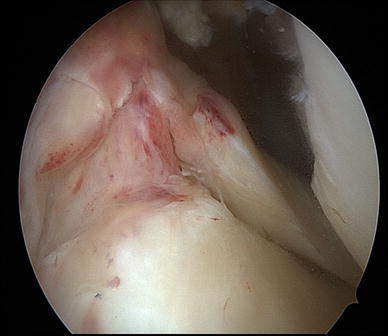
Arthroscopic view of acute lateral posterior root avulsion

At presentation two days after the injury, physical examination revealed signs of acute anterior laxity and pain was elicited over both lateral and medial joint lines. Full flexion was painful and restricted by knee swelling. Manual knee laxity tests, including the Lachman test, anterior drawer test and pivot shift test, were positive, as was the McMurray test. Measurement using a KT-2000 arthrometer (MEDmetric Corporation, San Diego, CA, USA) demonstrated a 5-mm side-to-side difference. Preoperative radiographic evaluation was normal. Magnetic resonance imaging (MRI) disclosed acute rupture of the ACL and tears in the roots of the posterior horns of both lateral and medial menisci (Fig. 1). Arthroscopic evaluation under regional anesthesia revealed an acute tear of the ACL at its midsubstance. Avulsion of the posterior root of the lateral meniscus was present in addition to acute avulsion at the posterior root of the medial meniscus with posterior displacement (Figs. 2, 3). No other intra-articular lesions were detected.

Treatment consisted of arthroscopic pullout suturing of both menisci and ACL reconstruction with an autologous bone-patellar tendon-bone graft. A transeptal approach was used for the medial meniscus root suture. The flat tip of an ACL guide (Arthrex, Naples, FL, USA) was introduced through the anterolateral portal at the footprint of the posterior root of the previously abraded medial meniscus. A tibial tunnel was made using a 2.9-mm guide pin from the anterolateral cortex of the proximal tibia to the footprint of the posterior root of the medial meniscus. Two nonabsorbable sutures were placed at the posterior root using a crescent-shaped suture hook. Both sutures ends were pulled out through the anterolateral cortex of the proximal tibia. The lateral meniscus was sutured in the same manner. The suture material for the lateral meniscus was pulled through the anteromedial cortex of the proximal tibia. The lateral and medial sutures were tied over two buttons after confirming sufficient reduction and tension.

**Fig. 4:**
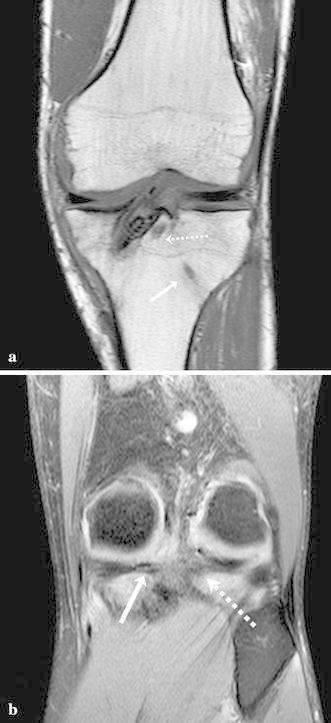
At MRI follow-up at three months, the coronal T1-TSE view (**a**) shows the tibial tunnel for ACL reconstruction and the tunnels for the medial root (*white arrow*) and for the lateral root (*dashed arrow*). **b** The coronal TSE-fat saturated MR image shows both medial and lateral roots healed

Postoperatively, the knee was kept in full extension in a brace locked at 0° for four weeks. Passive motion was allowed after the first two weeks and active motion was restricted to 90° during the first four weeks. Partial weight bearing was permitted at four weeks postoperatively, followed by full weight bearing at six weeks. After six weeks closed-chain strengthening was begun, and full flexion exercises were allowed. During the second and the third month, strengthening exercises and hydrokinetic therapy were implemented. Running on a treadmill was started after two months and training on the field was permitted after four months. Six months postoperatively, the patient was able to return to play in an official match with full range of knee motion. At the last follow-up of one year, no meniscal signs and symptoms were present. Manual knee laxity tests, including the Lachman test, anterior drawer test and pivot shift test, were negative. The side-to-side difference was 0.4 mm, as measured by the KT-2000 manual maximal test. The postoperative MRI showed a good healing process of both roots (Fig. 4).

## Discussion

The unusual finding in this case was avulsion of both posterior meniscal roots with a concomitant acute ACL tear. Medial posterior root avulsion usually results from chronic degenerative meniscal tears [2, 8, 10, 19] and is seldom associated with posterior cruciate ligament tears [14]. According to the radiological literature, the incidence of meniscal root tear is 8–9.8 % [3, 6, 20], whereas the orthopaedic literature reports a wider range between 6.7 % and 12.4 % [1, 9, 10]. This discrepancy stems from difficulties in radiographic diagnosis and in defining meniscal root tear. Two subcategories of meniscal root tear can be distinguished: root avulsion from the tibial plateau and meniscal posterior horn tear within 1 cm from the root. These tears are biomechanically similar because they can disrupt the circumferential fibers of the meniscus resulting in failure of the hoop strain mechanism [9, 11, 12]. Following rupture, the ability to resist extrusion under axial loading is definitely lost [4, 13].

In the only case described to date of a radial tear in both roots [15] concurrent with a chronic ACL tear, Lee et al. [15] postulated that the mechanism of injury was involvement of the posterior lateral root together with an ACL injury. The medial radial tear in the posterior root was caused by forceful mechanical stress secondary to instability. In the present case, avulsion of both meniscal roots was associated with an acute ACL tear. From a review of the video recording, the mechanism of injury was seen to be clearly due to forceful rotatory stress. As postulated by Park et al. [20], anterior tibial translation in an ACL injury may pull the lateral meniscus forward, stripping the meniscofemoral ligament away the meniscus attachment. The mechanism of medial meniscus root injury is more difficult to explain. Due to external rotation, for stress associated with compression axial load, the posterior horn is impinged by the femoral condyle. Markolf et al. [16] have shown that the anterior tibial force and the external tibial torque during knee loading produce relatively high posterior horn attachment forces, presumably by impinging the medial femoral condyle against the posterior meniscal rim.

In our patient, both meniscal roots were refixed with a transtibial technique. The sequelae of a medial root avulsion left in situ or misdiagnosed is functionally equivalent to total meniscectomy with meniscal extrusion and rapid progression to knee arthritis [17, 18]; however, there is no consensus on the treatment of lateral meniscal root tear. The fewer lateral meniscal tears in chronic versus acute ACL tears have led to conservative treatment of such lesions [5]. There are several reasons justifying this approach: concomitant ACL reconstruction creates blood clots and joint stability, increased blood supply to the posterior horn in comparison to the lateral meniscal pars intermedia, and absence of definitive clinical complaints when a lateral meniscus tear is left in situ. So, a radial or complex (radial and longitudinal) tear that occurs within 1 cm of the meniscal attachment may be more likely to heal spontaneously [7, 22]. Spontaneous healing after avulsion of the lateral root is less probable. The lateral meniscal root has two distinct insertions: one is anterior and attached to the posterior aspect of the tibial intercondylar eminence, and the other is posterior to and confluent with the meniscofemoral ligament. When root avulsion occurs, the latter insertion probably inhibits spontaneous healing because of continuous traction by the meniscofemoral ligament during knee movements. Recently, Schillamer et al. [21] demonstrated that posterior horn avulsion of the lateral meniscus causes peak tibiofemoral contact pressure to increase from 2.8 to 4.2 MPa, but that the peak pressure returns to normal after repair to bone via a transtibial tunnel.

In this rare case of tears in the roots of the posterior horns of both menisci, concomitant with an acute ACL tear, radiological and clinical outcome after surgery confirmed good healing. Both the lateral posterior horn and the medial posterior horn need to be considered when planning ACL reconstruction.
